# Proposal of a novel Artificial Intelligence Distribution Service platform for healthcare

**DOI:** 10.12688/f1000research.36775.1

**Published:** 2021-03-26

**Authors:** Antti Väänänen, Keijo Haataja, Katri Vehviläinen-Julkunen, Pekka Toivanen

**Affiliations:** 1 School of Computing, University of Eastern Finland, Kuopio, Pohjois-Savo, FI-70211, Finland; 2 Department of Nursing Science, University of Eastern Finland, Kuopio, Pohjois-Savo, FI-70211, Finland

**Keywords:** Artificial Intelligence, AI Platform, Healthcare AI, AI Distribution

## Abstract

In this paper, we focus on presenting a novel AI-based service platform proposal called AIDI (Artificial Intelligence Distribution Interface for healthcare). AIDI proposal is based on our earlier research work in which we evaluated AI-based healthcare services which have been used successfully in practice among healthcare service providers. We have also used our systematic review about AI-based healthcare services benefits in various healthcare sectors. This novel AIDI proposal contains services for health assessment, healthcare evaluation, and cognitive assistant which can be used by researchers, healthcare service provides, clinicians, and consumers. AIDI integrates multiple health databases and data lakes with AI service providers and open access AI algorithms. It also gives healthcare service providers open access to state-of-the-art AI-based diagnosis and analysis services. This paper provides a description of AIDI platform, how it could be developed, what can become obstacles in the development, and how the platform can provide benefits to healthcare when it will be operational in the future.

## 1 Introduction

Implementing Artificial Intelligence (AI) methods in healthcare services is the key element for reducing healthcare costs and improving health outcome. This have been proven in several studies where benefits of AI methods have been compared with the traditional process ^1^. Even if there are proven benefits when implementing methods using AI into healthcare services there are also many issues which need to be taken into account if we would like to see AI to have a breakthrough in the field of healthcare. We need to proceed with AI as one technology of augmented intelligence solution where AI will be helping clinicians and healthcare decision makers providing more accurate diagnosis, predictions, and guidance. We can accept AI as a natural evolution in healthcare information technology (IT) only when all actors can see benefits of AI and these benefits can be identified and approved by all actors in healthcare processes. To provide better chance of success to AI evolution or even “revolution”, we must provide the possibility to healthcare services developers and researchers to access scientifically studied and validated state-of-the-art AI methods and health information databases to enhance healthcare processes and healthcare outcome. These AI methods and databases should be accessible transparently and without high costs or regulatory barriers. Some companies, such as GE Healthcare, are already working with AI platforms which make quick implementation of proprietary AI algorithms or third-party AI algorithms possible inside one platform ^2^.

Our novel AIDI platform will be designed to provide easy access interface for healthcare service providers, healthcare IT companies, and researchers for utilizing state-of-the-art third party or self-developed AI methods for specific healthcare use cases. It contains cloud-based AI services which can be integrated to other end user organizations’ healthcare service platforms with open application programming interfaces (APIs). These AI services can be used by clinicians or other healthcare stakeholders for providing support in decision making. AIDI can be used for new AI methods validation and evaluation purposes. The goal of the state-of-the-art AIDI platform is to provide more accurate and cost-effective diagnosis, predictions to healthcare, and give researchers and developers a platform to validate and evaluate new AI methods.

The paper is organized as follows. Section 1 provides an overview of the AI benefits in healthcare sector and rationale for the development of AIDI platform. Section 2 gives an architectural and technical overview of AIDI platform. Section 3 discusses use cases, benefits, and issues of AIDI platform development. Finally, Section 4 concludes the paper and sketches some future work.

## 2 AIDI architecture and technical overview

**Figure 1.**
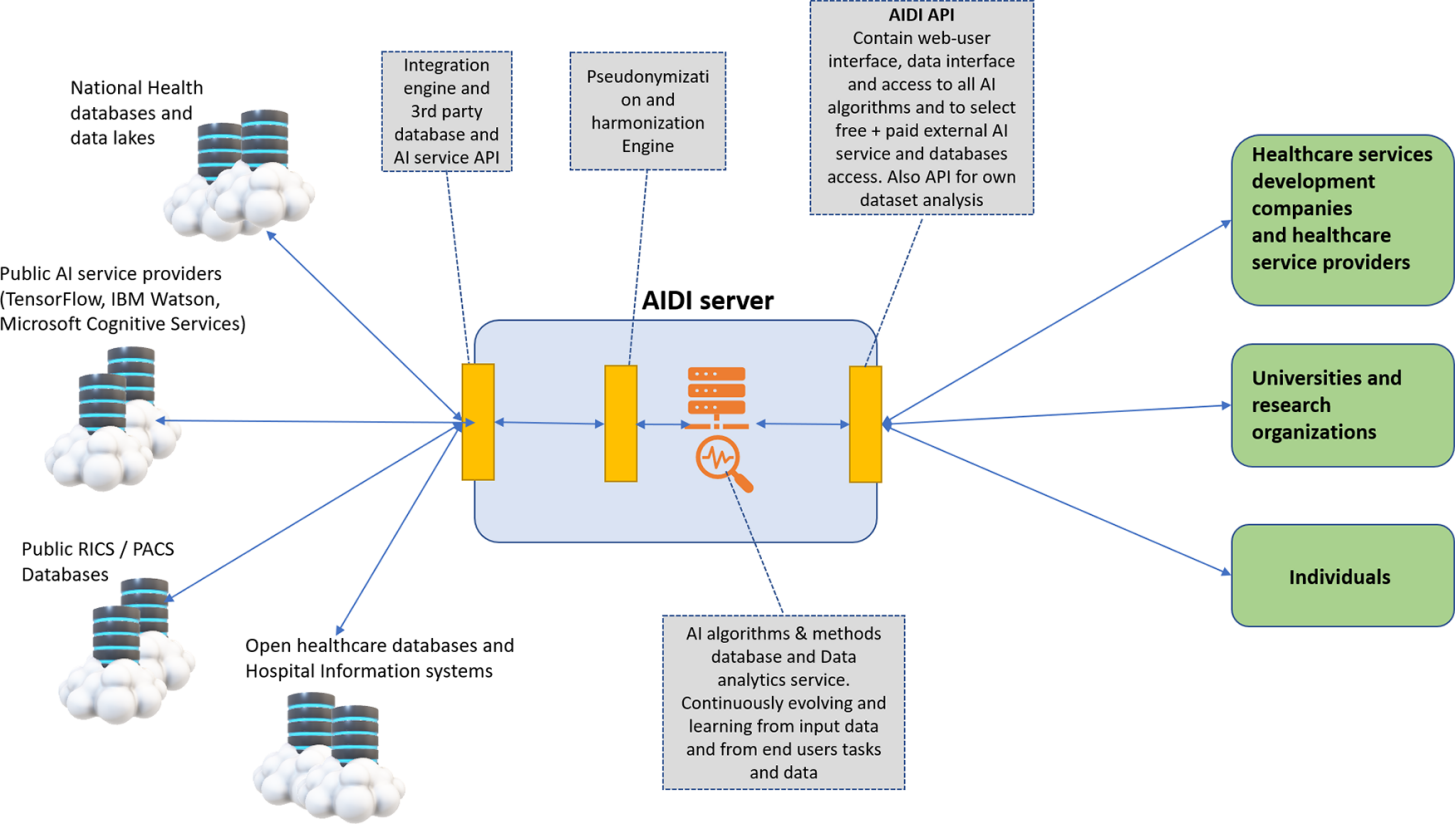
Basic architecture of AIDI.

In this section we provide an abstract architectural design of AIDI and details of its interfaces, application/software stack, and intended use. Figure 1 shows the basic architecture of AIDI containing actual server with data repository interfaces for data repositories and AI service providers, proprietary and public AI methods and algorithms database, and interface for end users. AIDI services will be operated in a cloud environment such as Microsoft Azure or similar.

**Figure 2.**
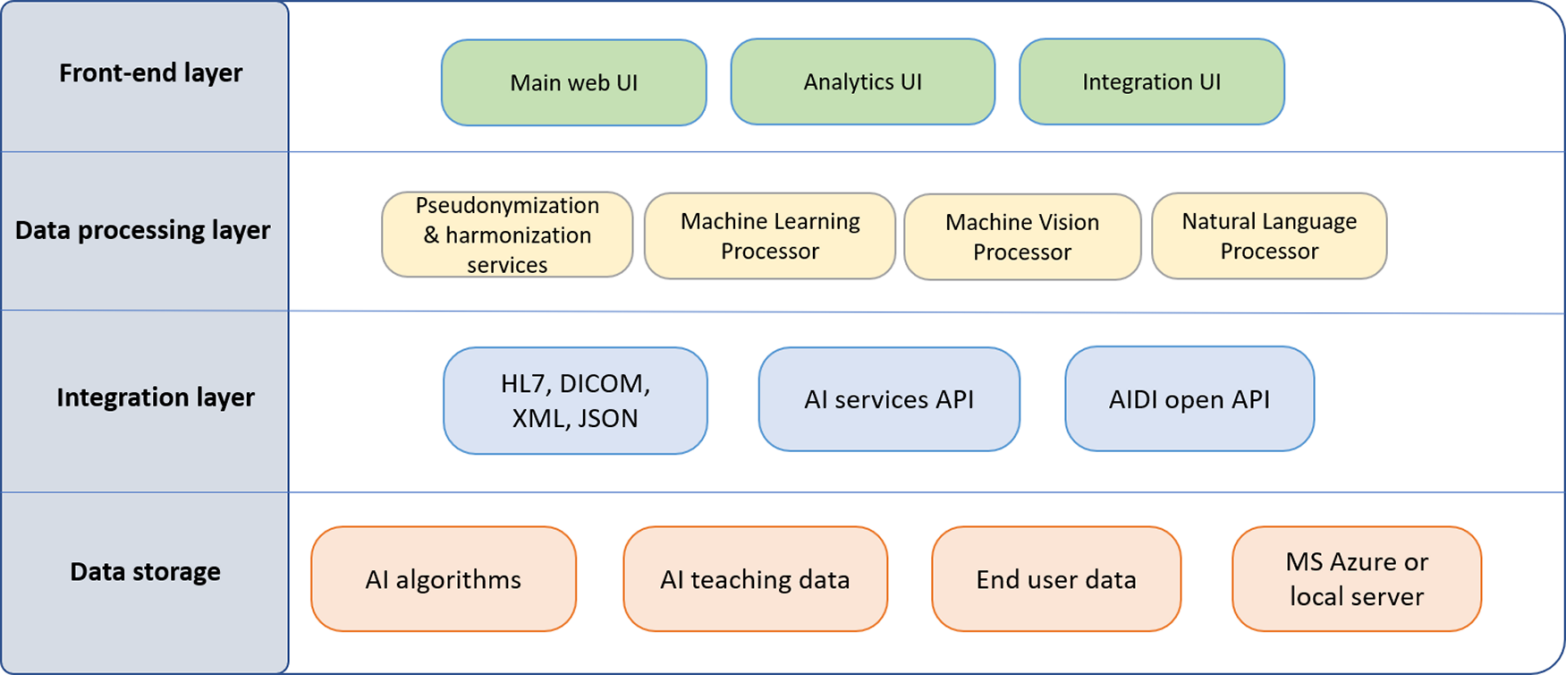
AIDI technology stack.

AIDI contains applications, service models, and data interfaces to end users, such as healthcare service development companies and healthcare service providers, researchers, and individuals. AIDI also has data interfaces to health databases, imaging databases, and AI service and algorithm providers. Figure 2 shows AIDI technology stack components.

AIDI technology is capable of parallel processing of vast amounts of data from multiple data sources and will provide real-time analytics for research purposes and clinical decision making. Technology stack modules will be developed by using basic software development tools and open source software modules. It contains four layers: Front-End, Data Processing, Integration, and Data Storage.

**Front-end** layer consists of web-based user interface (UI) for main application features, such as graphic tools and views of services and applications, selection of databases, analytics tools, and internal or third-party AI methods. Front-end contains also a separate analytics view where end users can see analytics of processed data and implement analysis view to own healthcare services or applications. It can be used by developers and researchers for own AI method testing and evaluation and comparison to other similar AI methods. Moreover, front-end contains UI for integration purposes where end users can configure own data integration settings, provide input data for AI, and implement own AI methods and data repositories. Front-end can be developed with Python and/or with ReactJS and data visualization can be enriched with streaming services visualization tools.

**Data processing** layer includes processing and analyzing methods for training data and user data with AI based processes. Pseudonymization and harmonization provide extra security layer to anonymization and harmonize data which can be received from multiple open or proprietary data lakes or data repositories. Machine learning processor can be used to perform data processing tasks, such as deep learning and supervised/unsupervised learning. Machine vision processor will provide AI, for example, image recognition and feature extraction needs. Natural language processor runs AI methods for language processing tasks, such as content extraction, classification, text generation, and machine translation. For data analysis and processing we can use third-party machine learning platforms, such as OpenAI ^3^, Tensor Flow ^4^, IBM Watson ^5^, and Azure Cognitive Services ^6^, and technologies, such as Python, and AI libraries, such as OpenCV, SciKit Learn, and Pandas.

**Integration layer** contains APIs for external databases, such as radiology imaging services (DICOM standard), hospital information systems (HL7 standard), and other third-party databases and data lakes (XML and JSON messaging formats). External data can be used as teaching data for AI methods. AI services API will provide integration to third-party AI algorithms and services, such as OpenAI, Tensor Flow, IBM Watson, and Azure Cognitive Services. AIDI open API will provide integration technology to end users to upload own data and AI methods to AIDI. For integration and data streaming AIDI can utilize streaming service platforms, such as Google Cloud Dataflow, Apache Kafka, or Amazon Kinesis.

**Data storage layer** has repositories for public and proprietary AI algorithms as well as database for AI teaching data retrieved from external databases or from end user database. End user database is used for maintaining users and organizations in AIDI services. AIDI has also service to import/export virtual machines and databases that AIDI can be easily installed to a cloud service, such as Microsoft Azure or to local server and database if AIDI would be used as proprietary service for specific company or organization use cases.

## 3 Use cases

We propose modular AIDI deep learning solution with AIDI’s own AI methods and third-party AI methods. As a starting point AIDI will provide AI methods for use cases which are used in prediction, diagnostic, and analytics. Robotic surgery and surgery live monitoring use cases are excluded due to the critical life-threatening nature of these use cases. AIDI solution will be able to analyze high volume data and distributed data and provide diagnosis or analysis from input data in real-time. Catalogue of AI methods can be expanded based on use cases arising from healthcare service providers or healthcare information system developers. We have evaluated that AIDI can include AI services which are explained in sections 3.1-3.9.

### 3.1 Automatic disease identification/diagnosis

Efficiency of AI has been identified in eye diseases ^7^, breast cancer ^8, 9^, and skin cancer ^10^ diagnosis when using data mining, natural language processing, and machine vision methods. Furthermore, glucose level prediction is one of the use cases where AI methods have been utilized successfully ^11^. AI methods can be used for tuberculosis diagnostics ^12^ and prediction of psychosis ^13^. AIDI platform can implement these studied AI methods to provide direct access to utilize these methods for customers and end users.

### 3.2 Personalised medicine

Personalised medicine is tailoring of medical treatment to the individual characteristics of each patient. The approach relies on the understanding of how a person's unique molecular and genetic profile makes them susceptible to certain diseases. Personalised medicine also focuses to provide detection of the diseases at an earlier stage when treatment activities can be applied more effectively ^14^. AIDI can monitor patient diagnosis and data and based on data provided to pre-trained AI methods AIDI can suggest most effective treatments and/or medication to patient.

### 3.3 Medication errors and medication non-adherence

AI methods to detect medication errors and increase medication adherence are used in healthcare sector. AI can be used to create screening system to detect potential medication errors and generate alerts to clinical decision support (CDS) systems and electronic medical record (EMR) systems ^15^. Machine vision and neural networks have been used efficiently to monitor medication intake by patients to enhance medication adherence. AIDI can provide access to these methods as a service to be utilized by healthcare service providers.

### 3.4 Identifying candidates for clinical trials

In clinical trials it is essential to identify potentially eligible patients to create more comprehensive clinical trials with lower costs. AI can be used to help in clinical trial design and to find patterns in large datasets for identifying most suitable patients before clinical trials start and AI can be used also for enabling more effective and accurate monitoring of patients during clinical trials. By using AI methods increase in enrolment in clinical trials has been observed. Furthermore, utilization of AI methods increases identification of eligible patients for clinical trials ^16^.

### 3.5 Epidemic outbreak prediction and management

On-going 2020 Covid-19 pandemic has increased the need to predict the progression of epidemics and pandemics. AI methods can be used for different epidemics by combining and analysing information of spatial spreading and behaviour data of the epidemic. As a result, estimation and prediction of epidemic spreading can be achieved ^17^. Real-time forecasting of epidemic outbreak with AI have been used in COVID-19 forecasting in China with high accuracy ^18^. Furthermore, AI methods can be used in managing on-going epidemic outbreaks by providing methods for identification of on-going epidemic outbreak, diagnosing and prognosing infections, and identifying possible therapeutic options ^19^. AIDI methods can provide end users the solutions which utilize data from public and private health databases and data lakes to predict possibility for epidemics.

### 3.6 Medical image diagnostics

AI has been used in connection with medical imaging for a long time and the use of AI methods has been extensively studied and proven to surpass human diagnosis in practical clinical work. In the field of medical imaging the AI technologies, such as DNN and DL, can produce remarkable improvement in healthcare outcome and they have proven to provide enhancement in speed, accuracy, and cost reduction in interpretation of medical images ^20^. Medical image diagnostics can be utilized in healthcare for multiple cases, such as for identifying cardiovascular abnormalities, detecting common eye diseases by optical coherence tomography, detecting musculoskeletal injuries, identifying neurological diseases, identifying thoracic complications, and in oncology for screening common cancers ^21‐23^. AIDI can provide these validated methods to end users for instant image analysis. Our plan is to provide AI methods for medical image diagnostics as among the first of AIDI services.

### 3.7 Health monitoring and preventive health

AI utilization in health monitoring and preventive health consist of solutions for continuous health status monitoring, healthcare assessment tools, and symptom checking solutions which can be used by patients and healthcare professionals periodically or continuously. AI is also commonly used in personal health monitoring and giving personalized suggestions for preventive health based on user parameters, such as activity tracking, nutrition, sleep analysis, weight, body composition, and vital signs, such as blood pressure and blood sugar. Personal health apps are the most common health monitoring solutions which collect data from user smartwatch and smartphone and utilize AI to provide analysis and suggestions for end users. There are also clinical remote monitoring solutions which utilize AI, such as cardiology monitoring, sleep monitoring, and posture monitoring ^24, 25^. AIDI can provide periodical/intermittent analytics from the collected data and provide analysis to healthcare services development companies or research organizations.

### 3.8 Virtual nursing assistants

Healthcare services utilize AI based virtual nursing assistants in various use cases. By using virtual nursing assistants, hospitals will be able to reduce sudden hospital visits and reduce the load of healthcare professionals. Virtual assistant applications can listen, talk, and give advice/recommendations based on patient conversation, health history and health data of the patient. Common virtual nursing assistant solutions provide pre-diagnosis for patients and healthcare professionals based on the healthcare assessment questionnaire and patient history data before entering the primary care ^26, 27^.

### 3.9 New AI methods evaluation

AIDI can be used to validate usability of new AI methods. AIDI will contain a growing number of AI methods for healthcare use cases. AIDI also provides access to training data and actual data. New AI methods can be evaluated and validated by comparing new AI method outcome (i.e., identification of accuracy, specificity, or sensitivity) and compare results with previous AI methods results. With this evaluation and validation service third-party AI method developers can create better AI methods which can be utilized in AIDI platform by companies or research organizations. Evaluation can be done by a specific multidisciplinary team and by scoring system in AIDI. This scoring provides end users valuable information about usability of selected AI method in specific use case.

**Figure 3.**
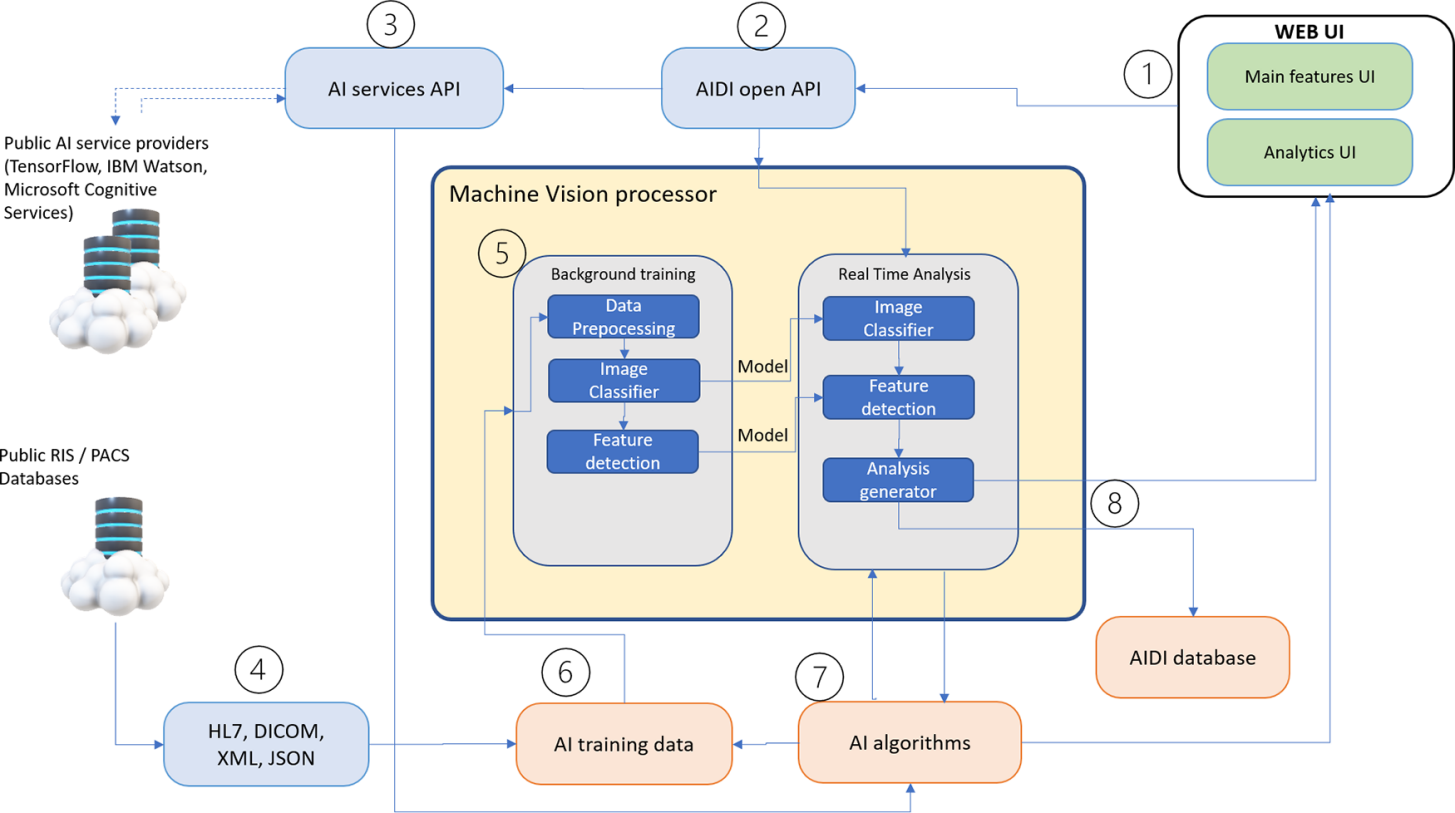
Automatic image diagnosis - process flow.

**As an example of AIDI** we present the process flow of use case where clinicians want to analyse radiology image to identify possible lesion from the image. Steps are explained and shown in Figure 3:

1. User sends radiology image to AIDI with information about use case (example case breast cancer analysis).
2. Open API module analyses query and transmits information and asks for details from the user about the use case.
3. AI services API select used AI service from data processing layer and select suitable algorithm from AI algorithm database or from third-party AI methods. In this example, selected service will be Machine Vision Processor and selected AI method is feature extraction.
4. AIDI Machine Vision Processor continuously receives radiology images for diagnosis from imaging databases for AI algorithm training.
5. Machine Vision processor uses training images for data pre-processing, classification, and feature detection.
6. Training data for AI processor is received from RIS/PACS systems as background service and AI algorithms are continuously trained with collected data.
7. Suitable AI algorithm for image analysis is selected and sent to Machine Vision Processor for real-time analysis.
8. Results and findings from image analysis will be sent back to user and will be shown to user in Analytics UI. AI algorithm may also be altered during real-time analysis and sent as new enhanced version of the algorithm into the AIDI database.
9. User will send diagnosis back to AIDI which can be used to improve the used AI algorithm accuracy. AIDI will monitor continuously the user-experienced diagnosis accuracy compared to diagnosis provided by AIDI.

**Advantages for actors in healthcare**

When utilizing AIDI in healthcare use cases we can provide immediate benefits for all actors in care process and service development. **For software and services development,** there is less time used for training AI methods, since AIDI developers can use ready-made AI methods and modify methods and algorithms to meet their own use case specific needs. There is also no need to search for training data for AI algorithms, because AIDI contains interface for training data repositories. **For healthcare service providers and caregivers,** there is instant prediction, analysis, or pre-diagnosis possibility with the software which utilize AIDI platform features. AIDI can provide enhancement in diagnosis accuracy and diagnosis speed. **For patients, healthcare customers, and people** who are tracking their wellbeing, AIDI provides accurate and instant diagnosis, prognosis, and predictions based on personal health data and verified and trained AI analytics from large datasets.

The savings potential when using AI in healthcare is very high. In USA annual savings are expected to be 150 billion USD by 2026 ^28^. When comparing this savings potential to total US healthcare expenditure in 2018 (3600 billion USD with 4,6% annual growth) we expect that US healthcare expenditure will be 5150 billion USD in 2026 ^29^. With these values we evaluate that AI utilization can bring 2,9% reduction in healthcare expenditure in the US. We evaluate that same cost reduction percentage can be achieved also globally.

AIDI benefits can be presented by the situation where state-of-the-art AI methods are integrated into AIDI and these services are utilized in healthcare in full scale. Benefits can be introduced in values, such as improvement in care outcome, cost reduction in healthcare, quality-of-life improvement among patients, and reduction in workload among healthcare professionals ^30^.

For evaluation of AIDI methods in healthcare we also suggest a first version of the weighted scoring system for AI benchmarking. Our novel scoring provides benefit assessment for evaluating the implementation of new AI method in certain healthcare use case. Output is informed as AIscore. Same tool with modified parameter information can be used to evaluate AI method benefits in other business sector use cases. Parameters of the equation are: Scientifically Proven Improvement compared to traditional method (SPI), Training Data Score (TDS), Cost Reduction (CR), Quality-of-Life improvement (QoL), and Workload Reduction (WR). Parameters and weights will be inserted by evaluation team. Evaluation team has researchers and professionals in a particular field of healthcare. Parameter values inform parameter impact, effect, positive outcome, or improvement in certain use case of AI method. For example, parameter SPI can be set by specificity and sensitivity of AI method. Furthermore, parameter TDS can be set by evaluation training data quality and amount of training data parameters for the specific healthcare use case. Parameters CR, QoL and WR will be scored by evaluation team. Each of the parameters will be using weighted coefficient (W _n_) based on the importance of parameter for specific use case. There is also acceptance parameter (AP) expressing whether method can be used for specific use case or not. AIscore will be developed and evaluated in future AIDI research projects.

By using AIDI and its validation of AI methods by AIscore, we can provide centralized AI distribution and utilization platform where all actors can benefit from effective and evaluated AI services. AIDI platform can also contain sales channel for evaluated AI methods which can be easily adopted for healthcare industry purposes. AI method developers can provide methods through AIDI as “Google Play” style where AI will be openly available.

**Concerns of AIDI adoption in healthcare**

Although AI methods in AIDI can provide scientifically proven enhancement in diagnosis and predictions accuracy, we must keep in mind that AIDI will only provide information and guidance for healthcare decision makers. AIDI platform or AI methods overall are not yet allowed to provide direct diagnosis. Healthcare professionals will always be responsible for providing diagnosis and final decision. There are also challenges which can emerge when AIDI services are utilized in healthcare. These issues are market acceptance and end user acceptance. Possible reasons for these issues are arising from the lack of curated healthcare data. Moreover, previous studies show that there might be resistance from healthcare professionals and patients to trust AI based decisions and diagnosis. Furthermore, people think that there is less “human touch” in care process when AI takes a greater role in healthcare. Moreover, shortage of regulations in AI in healthcare and data privacy can cause challenges. All these issues and challenges should be considered in planning phase of the AIDI project to enable acceptance of AIDI services to all end user groups and to all regulatory authorities.

## 4 Conclusion and future work

Based on our own research work related to AI benefits in healthcare and promising results from other researches in healthcare AI we can conclude that AI is beneficial to all actors in healthcare sectors. This “revolution” of AI in healthcare has not gone unnoticed by healthcare services development companies. Technology companies are continuously developing and releasing new AI services and even service platforms. Some of the platforms have similarity in AI methods distribution to our AIDI proposal. There are technology giants, such as IBM, Google, Microsoft, General Electric, and Siemens investing and developing AI solutions. We have evaluated that the drivers of the growth in healthcare AI development are based on scientifically proven advantages in care outcome and savings in healthcare costs with AI ^30^. These factors have created the very high market growth. Also, other factors driving the market growth are the increasing volume of healthcare data and growing complexities of datasets driving the need for AI, the intensifying need to reduce towering healthcare costs, improving computing power and declining hardware costs, growing number of cross-industry partnerships and collaborations, and rising imbalance between health workforce and patients driving the need for improved healthcare services. Furthermore, one major factor growing the AI market growth is the adoption of AI by multiple pharmaceutical and biotechnology companies across the world. Global healthcare AI market is estimated to grow from 4,9 billion USD (estimated market size in 2020) to 45,2 billion USD in 2026 giving 44,9% of compound annual growth rate for this 6-year period ^31^.

Our future research work will focus on continuing this research with AIDI platform development and its features evaluation with healthcare used cases. First AI services provided by AIDI will concentrate on image diagnostics. This decision is based on our findings during this research where we noticed that healthcare service providers need methods which can easily be implemented into medical imaging and image diagnostics services. AI-based imaging and machine vision methods are widely adopted and accepted by healthcare professionals and companies who develop image diagnostic solutions for healthcare. Furthermore, we will expand AI methods database as well as provide more integrations to training data and third-party AI service providers. We will also analyse business potential and healthcare organizations acceptance of AIDI platform based on the market feedback and implement suitable AI methods for AIDI platform based on market needs. Moreover, we will apply funding for a new scientific project to bring AIDI platform alive.

## Data availability

No data are associated with this article.
