# Supplementary material for: Proposal of a novel Artificial Intelligence Distribution Service platform for healthcare
Source: F1000Res. 2021 Mar 26;10:245. [Version 1] doi: 10.12688/f1000research.36775.1 (PMC8577055; doi:10.12688/f1000research.36775.1)
Supplement: Peer review history

# Peer review: Reviewer response for version 1

**DOI:** 10.5256/f1000research.39838.r97304

**Published:** 2021-11-08

**Authors:** Lorenzo Faggioni

**Affiliations:** 1 Diagnostic and Interventional Radiology, Department of Translational Research, University of Pisa, Pisa, Italy

Interesting, quite exhaustive manuscript aimed at presenting a novel AI-based service platform proposal called AIDI (Artificial Intelligence Distribution Interface for healthcare).

 I think that it could be further improved as follows:

- Please avoid mentioning commercial names or companies, unless indispensable to properly describe the data presented (e.g. GE Healthcare in the Introduction, Microsoft Azur at the beginning of Section 2).
- The use cases from 3.1 and 3.8 are well structured and give a brief but systematic account of the potential and actual uses of AIDI. However, I would strongly suggest expanding each of them by mentioning the main literature references related to each use case in a dedicated table (reporting article title, first author, publication date, use case topic and a short summary of the methods and main findings). This would add significant value to the manuscript.
- Use case 3.6 (Medical image diagnostics). A big contribution of AI in the field of medical imaging that should be mentioned is the possibility not only to detect disease conditions (e.g., cancer), but also to provide insights about patient outcome and eligibility to some given treatments or not (e.g., molecular target therapies). Please elaborate on this.
- The paragraph on "Concerns of AIDI adoption in healthcare" is an important one, yet it should be supported by literature references (which are currently lacking at all). Some useful references on this topic include (but are not limited to) include the following: 1) Coppola F., *et al*, doi 10.3389/fpsyg.2021.710982; 2) Triberti S., *et al*, doi 10.3389/fpubh.2020.00117

Is the topic of the opinion article discussed accurately in the context of the current literature?

Yes

Are arguments sufficiently supported by evidence from the published literature?

Partly

Are all factual statements correct and adequately supported by citations?

Yes

Are the conclusions drawn balanced and justified on the basis of the presented arguments?

Yes

Reviewer Expertise:

Radiology, Imaging informatics, Artificial Intelligence

I confirm that I have read this submission and believe that I have an appropriate level of expertise to confirm that it is of an acceptable scientific standard.

## References

[1] : Human, All Too Human? An All-Around Appraisal of the. *Front Psychol* .2021;12: 10.3389/fpsyg.2021.710982 710982 10.3389/fpsyg.2021.710982 34650476PMC8505993

[2] : A. *Front Public Health* .2020;8: 10.3389/fpubh.2020.00117 117 10.3389/fpubh.2020.00117 32140456PMC7043312

[3] : Imaging biomarkers in upper gastrointestinal cancers. *BJR Open* .2019;1(1) : 10.1259/bjro.20190001 20190001 10.1259/bjro.20190001 33178936PMC7592483

# Peer review: Reviewer response for version 1

**DOI:** 10.5256/f1000research.39838.r92571

**Published:** 2021-09-09

**Authors:** Marko Jäntti

**Affiliations:** 1 CEMIS (Center for Measurement and Information Systems), Kuopio, Finland

This paper aims at developing a service platform AIDI (Artificial Intelligence Distribution Interface for healthcare) and showing its main components. The study focuses on explaining the structure of the artifact that authors have designed. The main artifact is an AI-based service platform. The structure of the paper is clear and the topic is very up to date and interesting. However, there are several issues that could have been improved.The Authors can consider them as improvement ideas.

**Introduction** 

 The study refers to earlier work but does not provide a clear reference to those studies. 

 "proposal is based on our earlier research work in which we evaluated AI-based healthcare services".  Related to the above mentioned comment, the authors could have positioned their paper to previous academic studies and results more clearly. What does it mean in terms of this paper? For example, the title of the paper refers to "novel" platform. Positioning could have helped authors to identify and communicate better the elements that actually are "novel".

 There are existing case studies that utilize already cloud & AI services ( https://customers.microsoft.com/en-us/story/1399290856490209110-region-hovedstaden-health-provider-azure-en-we) and there are academic studies that mention Azure AI (R. Singh and N. Sharma, "Machine Learning based Medical Information Analysis, Estimations and  Approximations over Present Health Research Domain). Therefore, 'novel' parts could have been discussed further. 

**Section 2**

 The section 2 describes the basic architecture of AIDI and four layers: Front end, data processing, integration and data storage. From a software development point of view, the selection of these layers is obvious and clear, however,  one should ask 1) why these elements were chosen to be part of the artifact and 2) how the artifact (and opinions) have been designed /constructed by using the method. Transparency of this design / construction process is crucially important.

 How authors could have addressed this better? If authors continue using a systematic review ("We have also used our systematic review about AI-based healthcare services") , there are guidelines how to apply the methodology. See for example: https://libguides.library.curtin.edu.au/systematic-reviews. Authors are encouraged to look this when they prepare their next systematic reviews.

**Section 3**

 Use cases is from a reviewer's perspective the most mature and interesting part of the article focusing on usage scenarios of AIDI platform. Authors cite existing AI and Healthcare-related literature showing the use cases for utilizing AI in healthcare. Literature presented here provides strong support for using AI components for example in predicting and governing pandemic outbreaks and scanning large datasets for identifying & selecting patients for clinical trials. 

 Are the conclusions (opinions) drawn balanced and justified on the basis of the presented arguments? Authors could have followed the survey methodology in a more transparent way (defining exclusion/inclusion criteria and showing clearlier where their conclusions are based on?).

 There is certainly always place for improvement in papers but as a whole, AI-based healthcare platforms deserve more attention and this paper manages to contribute to the field by describing the architectural elements of AI-powered  healthcare platforms as well as usage scenarios derived from the literature.

Is the topic of the opinion article discussed accurately in the context of the current literature?

Yes

Are arguments sufficiently supported by evidence from the published literature?

Yes

Are all factual statements correct and adequately supported by citations?

Yes

Are the conclusions drawn balanced and justified on the basis of the presented arguments?

Partly

Reviewer Expertise:

Computer science

I confirm that I have read this submission and believe that I have an appropriate level of expertise to confirm that it is of an acceptable scientific standard.
